# Translation and validation of the Arabic version of Mindful Attention Awareness Scale (MAAS) among the Lebanese population

**DOI:** 10.1371/journal.pone.0310272

**Published:** 2024-12-11

**Authors:** Aya Taleb, Ali Ismail, Linda Abou-Abbas

**Affiliations:** 1 Neuroscience Research Center, Faculty of Medical Sciences, Lebanese University, Beirut, Lebanon; 2 Faculty of Medical Sciences, Lebanese University, Beirut, Lebanon; 3 INSPECT-LB, Institut National de Santé Publique, Epidémiologie Clinique et Toxicologie, Faculty of Public Health, Lebanese University, Beirut, Lebanon; Universiti Malaya, MALAYSIA

## Abstract

This study aimed to evaluate the psychometric properties of the Arabic version of the MAAS (A-MAAS) among Lebanese adults, determine its reliability and validity, and explore its associated factors. A cross-sectional study was conducted from August to December 2023. A sample of 483 Lebanese adults, aged between 18 and 60 years, from all the Lebanese governorates completed a questionnaire consisting of three sections: socio-demographic characteristics, health-related questions, and the Arabic versions of the Mindful Attention Awareness Scale (A-MAAS) and the Positive and Negative Affect Schedule (PANAS). Our findings revealed that the A-MAAS exhibited strong internal consistency (Cronbach’s alpha = 0.932). Confirmatory factor analysis (CFA) confirmed the one-dimensional structure accuracy of the scale, and the significant correlations with PANAS scales supported its convergent validity. Test-retest results displayed strong reliability (ICC = 0.983). The study revealed significant associations, indicating that worse memory performance, smoking, and negative emotions were linked to lower A-MAAS scores, suggesting lower levels of mindfulness. Our study contributes to the growing body of evidence supporting the utility of the MAAS in assessing mindfulness and attention. Continued research efforts will be crucial for advancing our understanding and application of mindfulness assessment tools in diverse populations and clinical settings.

## Introduction

Delving into the complexities of the human mind, consciousness unfolds as a rich amalgamation of awareness and attention [1]. While awareness provides a steady stream of background processing, attention refines this stream by pinpointing specific elements of experience, heightening their salience and impact on conscious perception [2, 3]. Traditionally, consciousness has been a phenomenon often taken for granted and not rigorously examined in Western scientific inquiry. However, in recent years, there has been a burgeoning interest in understanding consciousness and its implications for human cognition and behavior. This growing fascination has led to an exploration of various facets of consciousness, including its relationship to the human condition and its potential role in promoting well-being [4].

One area of particular interest within the study of consciousness is trait mindfulness. Coined by Brown and Ryan (2003), trait mindfulness refers to a fundamental state of consciousness that varies both within and between individuals. It is characterized by a heightened awareness and attention to the present moment, allowing individuals to fully engage with their experiences, thoughts, and emotions without judgment or reactivity [5]. Numerous recent studies have demonstrated that mindfulness can lead to various positive outcomes related to well-being. Recent meta-analysis highlighted the positive effects of mindfulness-based interventions on negative mental health outcomes such as depression, anxiety, stress, and burnout, as well as on positive psychological outcomes like life satisfaction. Additionally, mindfulness interventions have been associated with improved emotional intelligence, resilience, and job performance [6, 7]. While it is acknowledged that most individuals possess the innate ability to attend and be aware, there exists variance in individuals’ inclination and ability to sustain attention in the present moment. This capacity for mindfulness, while present in most individuals, exhibits significant variance both between and within individuals, and is influenced by a myriad of factors ranging from personality traits to environmental stimuli.

Developing psychometrically sound scales to capture both inter-individual and intra-individual differences in mindfulness is crucial. Various scales, such as the Mindful Attention Awareness Scale (MAAS) [5], Attention Control Scale (ATTC) [8], and Moss Attention Rating Scale (MARS) [9], have been developed. These scales enable researchers and clinicians to delve into the nuances of mindfulness and attention, ultimately contributing to a better understanding of their role in mental health and well-being.

The MAAS, pioneered by Brown and Ryan in 2003, stands out as a widely-used instrument for measuring mindfulness in everyday experiences, comprising 15 items that capture instances of attention fluctuations or declines in attentional capacity [5]. Its popularity has led to translations into several languages including French [10], Spanish [11], Chinese [12], and Mexican [13], demonstrating good validity and reliability across diverse cultural contexts. Moreover, the MAAS has been validated in various clinical populations, such as individuals with first-episode psychosis [14], Iranian women with breast cancer [15], and Iranian substance abusers [16], highlighting its utility across different clinical settings. However, the absence of an Arabic version of the MAAS poses challenges for its use in non-English-speaking regions such as the Arab World.

This study aims to assess the validity and reliability of the Arabic version of the Mindful Attention Awareness Scale (MAAS). Additionally, the study seeks to explore associated factors contributing to decreased mindfulness among the Lebanese population. The implications of this study are far-reaching, as the findings could inform the development of targeted interventions aimed at improving cognitive health and quality of life among the elderly population in Lebanon. By identifying factors associated with mindfulness, healthcare providers and policymakers can implement preventive measures and interventions to mitigate the impact of cognitive disorders and promote healthy aging in the Lebanese community.

## Methods

### Translation process

Permission to translate the Mindful Attention Awareness Scale (MAAS) into Arabic was obtained from the corresponding author [5]. The translation process followed the five steps outlined by Beaton et al. [17]. Initially, two independent bilingual native-Arabic translators translated the 15 items of the MAAS from English to Arabic, emphasizing clear and understandable language for the Lebanese population while avoiding literal translations. Any discrepancies between the translations were resolved through discussion and consultation with an expert in the field. Subsequently, the translated version was back-translated into English by two different bilingual translators unfamiliar with the original questionnaire to validate its accuracy. A comparative analysis between the back-translated version and the original MAAS was conducted to confirm semantic equivalency. Additionally, a panel of experts, including bilingual professionals and cultural experts, evaluated the face and content validity of the A-MAAS. Their feedback and recommendations were incorporated to finalize the translated questionnaire. Each item of the scale was rated on a 4-point Likert scale by the committee, with scores of 3 or 4 indicating relevance. The content validity index (CVI) was calculated based on the percentage of experts who rated each item as three or four. All items received a CVI of at least 0.8, ensuring their retention in the final version of the A-MAAS.

A pilot study was conducted to assess the clarity and comprehensibility of the translated items in the pre-final version of the questionnaire. A random sample of 15 healthy Lebanese participants was selected for this purpose. Participants were asked to provide feedback on any items they found unclear or confusing. Following the pilot study, no issues were reported, and participants did not suggest any changes to the scale. Therefore, no modifications were made to the questionnaire based on the findings of the pilot study, indicating that the translated items were deemed clear and comprehensible by the participants.

### Study design and participants

A cross-sectional study was conducted among healthy adult Lebanese individuals from various governorates throughout Lebanon between August 1, 2023 and November 30, 2023. Participants aged between 18 and 60 were targeted, with participation being entirely voluntary. Data collection was facilitated through a Google Form online survey, distributed via "WhatsApp, Facebook, and Instagram" using a snowball sampling technique, allowing for broad outreach and a diverse sample.

Exclusion criteria were implemented to ensure the focus remained on the intended healthy adult population. Participants with a history or diagnosis of neurological or psychological disorders, such as depression, dementia, Alzheimer’s disease, or migraines, were excluded based on a yes/no question at the beginning of the questionnaire. A "yes" response prevented further participation in the study. Additionally, following data collection, any participant data falling outside the specified age range was systematically removed and excluded from the analysis.

### Ethical considerations

The study obtained approval from the scientific committee of the Neuroscience Research Center (NRC) at the Faculty of Medical Sciences of Lebanese University (ID: 226/2/2023). Prior to participation, online informed consent was obtained from each participant, ensuring they understood the voluntary nature of their involvement and their right to withdraw at any time. All data were collected anonymously and handled confidentially, adhering to the principles outlined in the Declaration of Helsinki [18].

### Data collection

A standardized questionnaire, designed to take approximately 10–15 minutes to complete, was developed in the Arabic language, which is the native language of Lebanon. The introductory page of the questionnaire provided a concise overview of the survey’s background and objectives, along with instructions for completion. The questionnaire consisted of three sections:

- Socio-demographic characteristics: age, gender, education level, marital status, and working status, health-related questions including height, weight, alcohol consumption, smoking status, sleep hours, physical activity, presence of any health challenges, and the use of any medications.
- **A-MAAS** is a self-reported questionnaire comprising 15 items (S1 File). It evaluates mindfulness through a sensitive awareness of present experiences. Participants respond to each item on a 6-point scale ranging from "Almost always" to "Almost never" (1 = Almost always; 2 = Very frequently; 3 = Somewhat frequently; 4 = Somewhat infrequently; 5 = Very infrequently; 6 = Almost never). Scores are calculated by computing the mean of the 15 items, with higher scores indicating higher levels of mindfulness [5].
- **Positive and Negative Schedule (PANAS)** [19] developed by three psychologists: David Watson, Lee Anna Clark, and Auke Tellegen, in 1988, to improve former approaches of measuring mood that had uncertain or arguable results and reliability [20]. It is a self-report questionnaire that is made up of two 10-item scales to measure both positive and negative affect. Each item is rated on a 5-point scale (1 = Not at all; 2 = A little; 3 = Moderately; 4 = Quite a bit; 5 = Very much), where the total score is calculated by finding the sum of the 10 positive items, then the sum of the 10 negative ones. Scores range from 10 to 50 for each subscale; a higher score on the positive scale indicates more of a positive affect, and a lower score on the negative scale indicates less of a negative affect [21]. The Arabic version of the PANAS was developed by Kimberly Kaye Asner-Self and Mais Al-Nasa’h in 2014. This version was specifically tailored for the Jordanian population, and consequently, it was adopted for use in the present study [22].

### Sample size calculation

Sample size guidance suggests that having 5–10 participants per scale item would be sufficient for establishing the validity and reliability of a scale [23]. Given that the MAAS comprises 15 items, the recommended number of participants for the study would range from at least 75 to 150 individuals.

### Procedure

Participants were informed that their participation was purely voluntary, and consent was obtained through an online informed consent form. Approval was solicited by asking participants a yes or no question at the beginning of the survey, ensuring they understood the study’s purpose and their right to withdraw at any stage without consequence. The online questionnaire was distributed via popular social media platforms such as Facebook, WhatsApp, and Instagram. No financial incentives were offered for participation. Test-retest reliability of the Arabic version of the MAAS was evaluated by having 30 participants complete the scale twice, with a two-week interval between administrations.

### Statistical analysis

Statistical analysis was performed using the Statistical Package for Social Sciences (SPSS) software version 26.0 and AMOS. In descriptive statistics, continuous variables were reported as means and standard deviations (SD) and categorical variables as frequency (n) with percentages (%). Exploratory factor analysis (EFA) and confirmatory factor analysis (CFA) were conducted to examine the factorial validity, and they were carried out separately for the A-MAAS using random split-half samples. EFA was done on the first random-half sub-sample to investigate the factor structure of the A-MAAS using principal components analysis with varimax rotation. Kaiser-Meyer-Olkin (KMO) measure and Bartlett’s test of sphericity were used to assess the sampling adequacy. The number of factors preserved in the scale was established based on eigenvalues greater than 1 and a visual inspection of the scree plot, and items were eliminated in case they had low communalities (<0.4), or high cross-loadings. Structural equation modeling was utilized to conduct CFA, and maximum likelihood was employed to assess how well the data fits the A-MAAS instrument’s factor structure. The internal consistency of the scale was evaluated using Cronbach’s alpha, where a coefficient greater than 0.7 indicates acceptable internal consistency. For test retest reliability, the correlation between the total scores of two administrations of the 30 participants was obtained by calculating the intra-class correlation coefficient (ICC; average measure). ICC values ranging between 0.40 and 0.59 are considered fair, between 0.60 and 0.74 are good, and between 0.75 and 1.0 are excellent [24]. To examine the gender invariance of A-MAAS scores, we conducted sub-group CFA using the second random-half sub-sample [25]. We assessed measurement invariance at the configural, metric, and scalar levels [26]. Invariance was detected if ΔCFI ≤ 0.010 and ΔRMSEA ≤ 0.015 or ΔSRMR ≤ 0.010 [25, 27]. We aimed to test for gender differences on A-MAAS scores using an independent-sample t-test only if scalar or partial scalar invariance were established. Based on the methodology proposed by Ward and Meade in 2023 [28], our dataset underwent a moderate screening process. The prior screening step was done based on the bogus items and instructed response items that was included in the google forms. Then, a post hoc analysis on the collected data was done using long-string computation as an effective tool for identifying invariant responses. Simultaneously, we employed Mahalanobis distance, along with even-odd consistency, as optimal measures to discern random responses within our dataset. All the statistical tests were two-sided, and the significance level was set at 0.05.

## Results

### Baseline characteristics of the study participants

Table 1 displays the baseline characteristics of the study participants. Out of the 483 participants, the majority were women, accounting for 73.9% of the sample. The average age was 28.7 years, with a standard deviation of 9.7. Most participants were university students (86.4%), with 47% being full-time workers, and 53.4% reporting being married or in a relationship. Furthermore, the majority of participants reported being non-smokers (74.3%). Approximately 53.2% reported sleeping between 4–7 hours per day, while 41.4% engaged in physical activity only occasionally. Additionally, 60.5% of participants did not report any underlying medical condition.

**Table 1:** Baseline characteristics of the study participants (N = 483).

Characteristics	All
	(N = 483)
**Age (years) Mean (SD)**	28.69 (9.75)
**Gender n (%)**	
Men	126 (26.1)
Women	357 (73.9)
**Educational level n (%)**	
Primary or no education	17 (3.5)
Secondary	44 (9.1)
University	422 (86.4)
**Marital status n (%)**	
Married/Partnered	210 (53.4)
Single	259 (53.6)
Divorced	14 (2.9)
**Employment status n (%)**	
Full time	227 (47)
Part time	57 (11.8)
Unemployed	199 (41.2)
**Smoking Status n (%)**	
Non smoker	359 (74.3)
Past smoker	9 (1.9)
Current smoker	115 (23.8)
**Alcohol consumption n (%)**	
No	467 (96.7)
Mild consumption	14 (2.9)
Severe consumption	2 (0.4)
**Sleep period (hour/day) n (%)**	
1–4 h/d	17 (3.5)
4–7 h/d	257 (53.2)
7–10 h/d	203 (42)
More than 10 h/d	6 (1.2)
**Physical activity n (%)**	
No	143 (29.6)
Sometimes	200 (41.4)
Frequently	140 (29)
**Number of underlying medical conditions n(%)**	
0	292 (60.5)
1	142 (29.4)
2 or more	49 (10.1)

N: frequency; SD: standard deviation; %: percentage; h/d: hour/day

### Psychometric properties of the A-MAAS

#### Reliability of the A-MAAS

Reliability of MAAS was measured in terms of internal consistency and test–retest reliability. The internal consistency results were analyzed using Cronbach’s alpha coefficients and composite reliability (CR) coefficients, which exhibited high internal consistency, with a Cronbach’s alpha of 0.932. The corrected–item to total correlation coefficients varied from 0.557 to 0.769. Cronbach’s alpha remained consistent across all items of the scale, ranging from 0.925 to 0.931, and the elimination of any individual item did not significantly affect its value (Table 2).

**Table 2:** Internal consistency of the A-MAAS.

Item-Total Statistics				
	Scale Mean if Item Deleted	Scale Variance if Item Deleted	Corrected Item-Total Correlation	Cronbach’s Alpha if Item Deleted
**A-MAAS 1**	56.43	257.520	0.666	0.928
**A-MAAS 2**	55.92	260.401	0.639	0.928
**A-MAAS 3**	56.28	256.390	0.712	0.926
**A-MAAS 4**	56.79	256.355	0.638	0.928
**A-MAAS 5**	56.43	262.030	0.557	0.931
**A-MAAS 6**	56.54	260.291	0.583	0.930
**A-MAAS 7**	56.47	251.187	0.762	0.925
**A-MAAS 8**	56.40	252.232	0.769	0.925
**A-MAAS 9**	56.66	255.487	0.684	0.927
**A-MAAS 10**	56.23	253.023	0.760	0.925
**A-MAAS 11**	56.99	260.703	0.603	0.929
**A-MAAS 12**	55.91	258.251	0.668	0.928
**A-MAAS 13**	57.43	260.703	0.563	0.931
**A-MAAS 14**	56.70	252.714	0.758	0.925
**A-MAAS 15**	56.13	255.322	0.646	0.928

N = 483; A-MAAS: Mindful Attention Awareness Scale–Arabic version

For the test-retest reliability, the correlation between the total A-MAAS scores of the two administrations was derived using the Intraclass Correlation Coefficient (ICC). The latter showed a strong reproducibility, with an ICC of 0.983 (CI = 0.965 to 0.992; p <0.001), indicating strong reliability.

#### Validity of the A-MAAS

After randomly dividing the sample size into two groups, each with n = 241, an exploratory factor analysis (EFA) was conducted on the A-MAAS scale using the first sample. The lowest communality value was that of the fifth item of the scale (A-MAAS 5) with a value of 0.366, and the highest was that of the eighth one (A-MAAS 8) with a value of 0.661 (Table 3). The analysis demonstrated a high KMO measure of 0.951, surpassing the commonly accepted threshold of 0.60 [29], indicating strong sampling adequacy. Additionally, Bartlett’s test of sphericity yielded a highly significant result (χ2 = 3901.814, df = 105, p-value < 0.0001), suggesting that the variables are significantly correlated and thus suitable for factor analysis. The scree plot of the eigenvalues illustrated a 1-factor structure for the MAAS scale, explaining 51.8% of the total variance.

**Table 3:** Exploratory factor analysis of the A-MAAS scale (N = 242).

	Communality
A-MAAS 1	0.508
A-MAAS 2	0.478
A-MAAS 3	0.576
A-MAAS 4	0.473
A-MAAS 5	0.366
A-MAAS 6	0.403
A-MAAS 7	0.648
A-MAAS 8	0.661
A-MAAS 9	0.535
A-MAAS 10	0.650
A-MAAS 11	0.431
A-MAAS 12	0.513
A-MAAS 13	0.380
A-MAAS 14	0.637
A-MAAS 15	0.488

A-MAAS: Mindful Attention Awareness Scale- Arabic version

**Fig 1:**
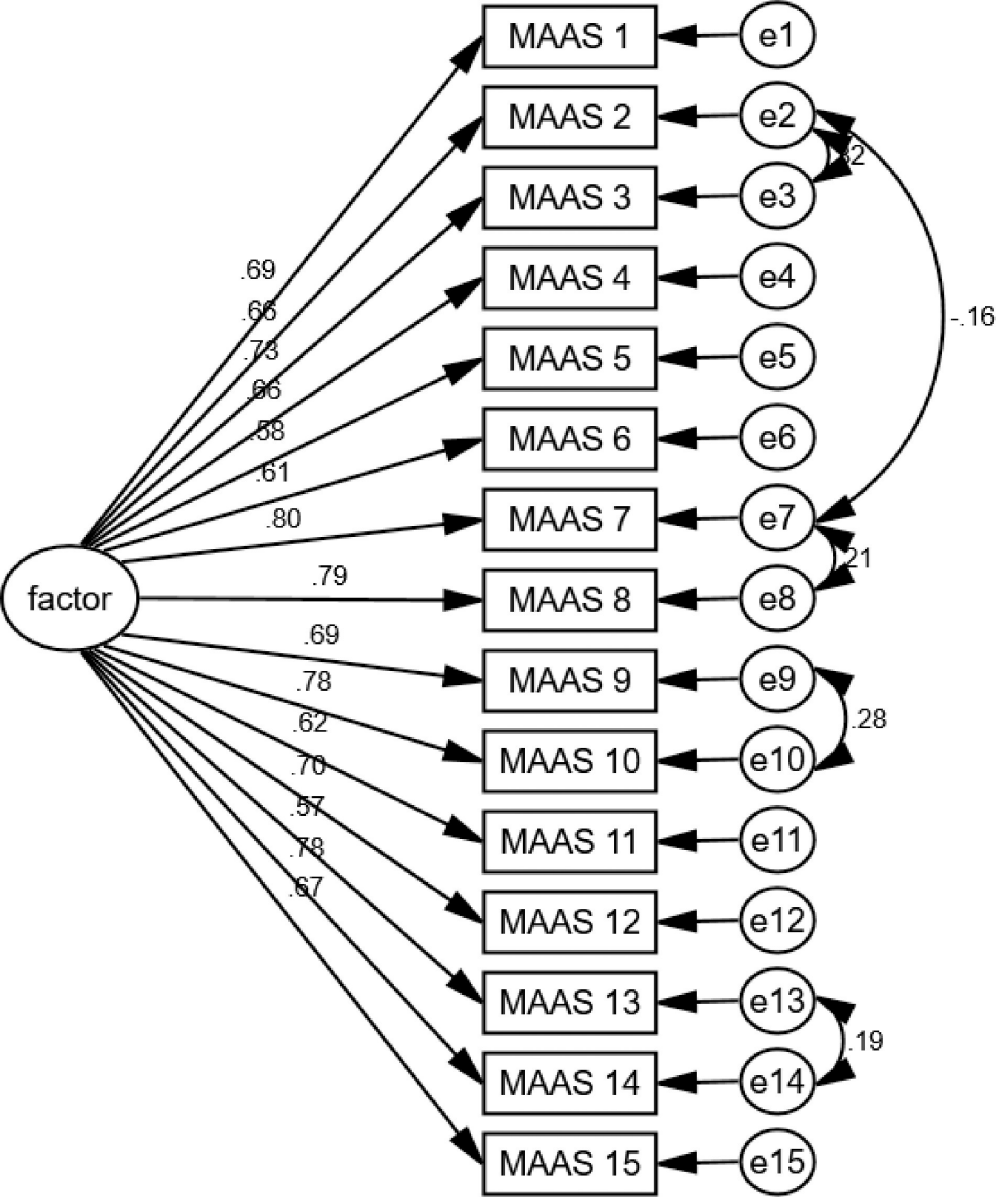
One-factor model of the Arabic version of the Mindful Attention Awareness Scale (A-MAAS) (N = 241).

After analyzing the other half of the sample size to confirm the unidimensional structure of the A-MAAS scale, as suggested by the exploratory factor analysis (EFA), we found that the model fit well with the data. The EFA indicated a 1-factor solution, which was confirmed by the inspection of the model. The goodness of fit statistics for the model were as follows: NFI = 0.901, CFI = 0.922 above the cut-off value 0.9, RMSEA = 0.084 considered medium, and χ2 / df = 4.395 which is acceptable [30]. All factor loadings were above 0.8 for each factor, indicating strong correlations between observed measures and latent variables. Moreover, all loaded items revealed significant correlations. Further analysis involved incorporating error covariance between specific pairs of items, resulting in a significant enhancement of the fit indices for the model. Notably, the CFI increased to 0.955, and the RMSEA decreased to 0.065, both indicating an improved fit for the model. All standardized factor loadings for the one-factor model were significant at p <0.01 (Fig 1).

The Spearman correlation analysis reveals significant associations between the MAAS score and PANAS subscales r = 0.125, P-value = 0.006 with PANAS positive score indicates that individuals reporting higher mindfulness levels also tend to exhibit elevated positive affect. Conversely, a negative correlation of r = -0.438, P-value<0.001 with PANAS negative score suggests that heightened mindfulness is associated with reduced negative affect.

#### Gender invariance

Gender invariance was rigorously assessed in the second random half subsample of the dataset. Table 4 indicates that the differences in Comparative Fit Index (ΔCFI) and Root Mean Square Error of Approximation (ΔRMSEA) were below the respective thresholds of 0.010 and 0.015 in model comparisons, suggesting strong evidence of configural, metric, and scalar invariance across gender. Furthermore, analysis within the second random half subsample revealed no statistically significant difference in A-MAAS scores between men (Mean = 62.14, SD = 18.29) and women (Mean = 60.03, SD = 16.65), with a t-value of 0.815 and a p-value of 0.416.

**Table 4:** Measurement invariance across gender in the second random split-half subsample.

Model	X^2^	Df	CFI	RMSEA	SRMR	Model Comparison	ΔCFI	ΔRMSEA	ΔSRMR	Δχ2	Δdf	p-value
**Configural**	450.6	170	0.931	0.058	0.126	-	-	-	-	-	-	-
**Metric**	454.5	184	0.933	0.055	0.139	Configural vs. metric	0.002	0.003	0.013	3.9	14	0.999
**Scalar**	485.5	198	0.929	0.055	0.139	Metric vs. scalar	0.004	0.000	0.000	31	14	0.00

#### Careless responding analysis

To ensure the reliability of participant responses, we meticulously scrutinized the potential for careless responding. Drawing from the methodologies proposed by Meade and Craig [31] and Curran et al. [32], we integrated an infrequency scale into our survey items to flag respondents who might have answered randomly or hastily. To evaluate the solidity of our dataset, we conducted a comprehensive post hoc analysis using a multifaceted approach advocated by Ward and Meade [28]. While implementing an ideal screening step before data collection proved unfeasible in our scenario, we implemented various strategies to detect potential issues in participant responses. Post hoc analysis of the collected data involved leveraging longstring computation as an effective tool for identifying invariant responses, particularly instances of extreme straightlining where respondents consistently selected the same option. Establishing a definitive cutoff value for longstring analysis posed challenges due to the absence of a specific threshold for excessively prolonged identical responses. Nonetheless, our dataset analysis yielded meaningful insights, with no responses exceeding the cutoff range of 6 to 14 and a maximum longstring value of 14 [33]. Additionally, employing the even-odd index methodology entailed segregating scale items into two subscales based on even and odd numbering. Both subscales demonstrated highly similar Cronbach’s alpha values, indicating consistent internal reliability. The significant correlation of 0.879 between these subscales, supported by a robust Spearman Brown coefficient of 0.936, suggested diligent completion of the questionnaire, surpassing the 0.30 threshold proposed by Jackson (1976) [34]. Furthermore, the Mahalanobis distance, introduced by Mahalanobis in 1936, served as a multivariate outlier statistic, considering the correlational structure between items. By determining the p-value corresponding to the Chi-Square value with 5 degrees of freedom, all responses exhibited a p-value above the threshold value of 0.001, as suggested by Tabachnick and Fidell [35], indicating the absence of outliers. This comprehensive approach ensures the robustness and reliability of our data, thereby enhancing the validity of our study outcomes.

#### Factors associated with A-MAAS

Table 5 displays the results of stepwise linear regression, revealing several significant associations with MAAS scores. Firstly, the EMQ-R score exhibited a statistically significant negative relationship with MAAS scores (Unstandardized Coefficient: -0.156, Standardized Coefficient: -0.103, 95% CI: -0.299 to -0.012, p = 0.034). Secondly, being a current smoker showed a significant negative association with MAAS scores (Unstandardized Coefficient: -4.106, Standardized Coefficient: -0.103, 95% CI: -7.008 to -1.205, p = 0.006). Additionally, PRMQ scores demonstrated a statistically significant negative correlation with MAAS scores (Unstandardized Coefficient: -0.616, Standardized Coefficient: -0.446, 95% CI: -0.730 to -0.501, p < 0.0001). Lastly, the PANAS Negative scale exhibited a significant negative correlation with MAAS scores (Unstandardized Coefficient: -0.378, Standardized Coefficient: -0.201, 95% CI: -0.559 to -0.198, p < 0.0001).

**Table 5:** Regression analysis of factor influencing A-MAAS scores (N = 483).

	Unstandardized Coefficients	Standardized Coefficients	95% Confidence Interval		P-value
			Lower Bound	Upper Bound	
**EMQ-R Score**	-0.156	-0.103	-0.299	-0.012	0.034
**Current Smoker vs non smoker**	-4.106	-0.103	-7.008	-1.205	0.006
**PRMQ Score**	-0.616	-0.446	-0.730	-0.501	<0.0001
**PANAS Negative**	-0.378	-0.201	-0.559	-0.198	<0.0001

Note: A-MAAS: Mindful Attention Awareness Scale-Arabic version; EMQ-R: Everyday Memory Questionnaire- Revised; PRMQ: Prospective and Retrospective Memory Questionnaire; PANAS: Positive and Negative Affect Schedule; Factors entered into the model: Age, Gender, Marital status, Education level, Smoking status, Physical activity, underlying medical condition, EMQ-R score, PANAS scores

## Discussion

In this study, we aimed to translate and assess the psychometric characteristics of the A-MAAS among a sample of 483 healthy Lebanese adults aged 18 to 60 years. Additionally, we sought to investigate factors associated with A-MAAS scores. Our findings indicate that the A-MAAS version demonstrated satisfactory psychometric properties within the targeted population. Furthermore, our analysis revealed associations between A-MAAS scores and attention, memory, smoking, and negative affect.

The robust reliability values obtained for the A-MAAS underscore its strong internal consistency and test-retest reliability, indicative of its efficacy in measuring mindfulness among Lebanese adults. The high Cronbach’s alpha coefficient of 0.932 suggests a high level of internal consistency, reaffirming the scale’s ability to accurately capture mindfulness-related constructs. These findings align with previous studies on the original MAAS and its adaptations in various cultural contexts, where similarly high reliability coefficients were reported. For instance, the Cronbach’s alpha values reported for the original MAAS version, the French adaptation, the Chinese version, the Mexican adaptation, and the Spanish adaptation all demonstrate strong internal consistency, ranging from 0.81 to 0.89 [5, 10–13]. This consistency across diverse cultural settings highlights the reliability and robustness of the MAAS as a tool for assessing mindfulness globally. Furthermore, the high intraclass correlation coefficient (ICC) value of 0.983 (95%CI: 0.965 to 0.992, p < 0.001) obtained in our study indicates excellent test-retest reliability, suggesting that A-MAAS scores remain stable over time.

As to construct validity, the Arabic version of the MAAS exhibited robust model fits for the 1-factor structure. This unidimensional model lines up with the original MAAS [5], the French version of the MAAS [10], the Chinese version [12], the Mexican version [13], and the Spanish one [11]. For convergent validity, the A-MAAS showed a statistically significant positive correlation with PANAS positive score and negative one with PANAS negative score. This indicates that individuals scoring higher on the A-MAAS also tend to report higher levels of positive affect and lower levels of negative affect, which supports its convergent validity.

The study’s finding of measurement invariance across gender for the A-MAAS indicates that the scale consistently measures mindfulness for both men and women. This suggests that any differences in mindfulness scores between genders likely reflect genuine variations in mindfulness levels rather than biases within the scale. This ensures that the A-MAAS is equally valid and reliable for assessing mindfulness in both men and women, facilitating meaningful comparisons between genders in research and clinical settings.

The findings suggest an association between memory and mindfulness, with better memory functioning associated with improved attention. Studies, such as Chun and Turk-Browne’s work, underscore the role of memory in directing attention, given the involvement of memory-related brain regions like the hippocampus and medial temporal lobe [36]. These observations align with established neuroscientific findings highlighting the interconnection between memory and mindfulness.

Furthermore, our results indicate that smoking is associated with lower MAAS scores, indicative of poorer capacity of smoker to concentrate on their present events to practice mindfulness. This is consistent with longitudinal research showing that smoking, particularly during adolescence, contributes to attention problems [37]. Another study stated that smokers have lower levels of mindfulness compared to non-smokers, suggesting that smoking has a negative effect on mindfulness [38]. Neuroimaging studies have also revealed greater age-related brain volume loss in smokers, particularly in areas crucial for mindfulness [39]. Addressing smoking cessation becomes crucial not only for overall health but also for mitigating attention-related challenges.

Additionally, our study showed that negative affective states can affect significantly the capacity to concentrate and to practice mindfulness. Research by Vuilleumier P. supports this, showing the impact of emotions on cognitive functions, particularly mindfulness [40]. Chronic stress, for instance, impairs mindfulness control and resource allocation precision [41]. These findings emphasize the importance of fostering an optimistic mindset and integrating positive habits into daily life to support cognitive well-being.

The study has some noteworthy limitations. Firstly, the reliance on self-reported measures introduces potential biases such as recall bias and subjective interpretation. The exclusion of participants with neurological or psychiatric disorders further restricts the generalizability of the results. Moreover, the convenience sampling technique introduces selection bias, impacting the external validity of the findings. Despite these limitations, the study provides valuable insights into the validity and cultural suitability of the MAAS among Lebanese individuals, offering informative data for mindfulness evaluation. Future research should aim to integrate both self-report and objective assessments to provide a more comprehensive understanding of attention functioning while addressing the limitations highlighted in this study.

The successful adaptation and validation of the Arabic version of the A-MAAS among Lebanese adults, alongside the exploration of factors associated with A-MAAS scores, carry significant implications. Firstly, it offers a culturally relevant tool for assessing mindfulness in Arabic-speaking populations, enabling researchers and practitioners to accurately measure and monitor mindfulness levels in this context. This adaptation not only facilitates cross-cultural research but also ensures that interventions targeting mindfulness are culturally appropriate and effective. Secondly, the study’s findings illuminate the interplay between mindfulness and various cognitive and emotional factors, including attention, memory, smoking, and negative affect. By identifying these associations, the study provides valuable insights into the complex dynamics of mindfulness in the Lebanese context. Such understanding can inform the design and implementation of interventions aimed at promoting mental well-being and addressing risk factors such as smoking and negative affect in the Lebanese community.

## Conclusion

Our findings underscore the validity and reliability of the Arabic version of the MAAS as a valuable tool for assessing mindfulness and attention functioning among Lebanese healthy adults. The identified associated factors suggest lifestyle habits that can improve attention, promoting better health and healthier aging. These include avoiding smoking, fostering optimism, and managing negative emotions and thoughts. The observed association between better memory and enhanced attention functioning suggests avenues for further exploration. Diverse populations and investigations into various clinical cases are essential to bolster the validity and applicability of the MAAS in assessing mindfulness and attention across different contexts. Our study contributes to the growing body of evidence supporting the utility of the MAAS in assessing mindfulness and attention, while also pointing towards actionable strategies for enhancing attentional abilities and overall well-being. Continued research efforts will be crucial for advancing our understanding and application of mindfulness assessment tools in diverse populations and clinical settings.

## Supporting information

S1 File MAAS tool English version. (PDF)
