# Supplementary material for: Translation and validation of the Arabic version of Mindful Attention Awareness Scale (MAAS) among the Lebanese population
Source: PLoS One. 2024 Dec 11;19(12):e0310272. doi: 10.1371/journal.pone.0310272 (PMC11633951; doi:10.1371/journal.pone.0310272)
Supplement: S1 File — (PDF) [file pone.0310272.s001.pdf]

**The Mindful Attention Awareness Scale (MAAS)**

The trait MAAS is a 15-item scale designed to assess a core characteristic of mindfulness, namely, a receptive state of mind in which attention, informed by a sensitive awareness of what is occurring in the present, simply observes what is taking place.

Brown, K.W. & Ryan, R.M. (2003). The benefits of being present: Mindfulness and its role in psychological well-being. *Journal of Personality and Social Psychology, 84,* 822-848.

Carlson, L.E. & Brown, K.W. (2005). Validation of the Mindful Attention Awareness Scale in a cancer population. *Journal of Psychosomatic Research, 58,* 29-33.

Instructions: Below is a collection of statements about your everyday experience. Using the 1-6 scale below, please indicate how frequently or infrequently you currently have each experience. Please answer according to what really reflects your experience rather than what you think your experience should be. Please treat each item separately from every other item.

| 1 | 2 | 3 | 4 | 5 | 6 |
|---|---|---|---|---|---|
| almost always | very frequently | somewhat frequently | somewhat infrequently | very infrequently | almost never |

_____ 1. I could be experiencing some emotion and not be conscious of it until some time later.
_____ 2. I break or spill things because of carelessness, not paying attention, or thinking of something else.
_____ 3. I find it difficult to stay focused on what’s happening in the present.
_____ 4. I tend to walk quickly to get where I’m going without paying attention to what I experience along the way.
_____ 5. I tend not to notice feelings of physical tension or discomfort until they really grab my attention.
_____ 6. I forget a person’s name almost as soon as I’ve been told it for the first time.
_____ 7. It seems I am “running on automatic,” without much awareness of what I’m doing.
_____ 8. I rush through activities without being really attentive to them.
_____ 9. I get so focused on the goal I want to achieve that I lose touch with what I’m doing right now to get there.
_____ 10. I do jobs or tasks automatically, without being aware of what I'm doing.
_____ 11. I find myself listening to someone with one ear, doing something else at the same time.
_____ 12. I drive places on ‘automatic pilot’ and then wonder why I went there.
_____ 13. I find myself preoccupied with the future or the past.
_____ 14. I find myself doing things without paying attention.
_____ 15. I snack without being aware that I’m eating.

Scoring: To score the scale, simply compute a mean (average) of the 15 items.
